# A qualitative analysis to identify the elements that support department level change in the life sciences: The PULSE Vision & Change Recognition Program

**DOI:** 10.1371/journal.pone.0217088

**Published:** 2019-05-30

**Authors:** Marcy Peteroy-Kelly, Loretta Brancaccio-Taras, Judy Awong-Taylor, Teresa Balser, Thomas Jack, Sara Lindsay, Kate Marley, Sandra Romano, J. Akif Uzman, Pamela Pape-Lindstrom

**Affiliations:** 1 Department of Biology, Pace University, NY, NY, United States of America; 2 Department of Biological Sciences, Kingsborough Community College-CUNY, Brooklyn, NY, United States of America; 3 Department of Biology, Georgia Gwinnett College, Lawrenceville, GA, United States of America; 4 Office of the Provost and Vice President of Academic Affairs, Dalhousie University, Halifax, Nova Scotia, Canada; 5 Department of Biological Sciences, Dartmouth College, Hanover, NH, United States of America; 6 School of Marine Sciences, University of Maine, Orono, ME, United States of America; 7 Department of Biology, Doane University, Crete, NE, United States of America; 8 College of Science and Mathematics, University of the Virgin Islands, St. Thomas, Virgin Islands, Territory of the United States of America; 9 College of Sciences and Technology, University of Houston-Downtown, Houston, TX, United States of America; 10 Division of Science, Technology, Engineering and Math, Harford Community College, Bel Air, MD, United States of America; Murray State University, UNITED STATES

## Abstract

The 2011 report, *Vision and Change in Undergraduate Biology Education*: *A Call to Action*, provided the impetus to mobilize the undergraduate life sciences education community to affect change in order to enhance the educational experiences of life sciences majors. The work of the appointed Partnership for Undergraduate Life Sciences Education (PULSE) Vision and Change (V&C) Leadership Fellows has focused on the development of programs and resources to support departmental change. In this report, we present a qualitative assessment of several documents generated from the PULSE V&C Leadership Fellow Recognition Team. The Recognition Team developed two initiatives to provide departments with feedback on their change process. The first initiative, the validated PULSE V&C Rubrics, enables departments to collaboratively self-assess their progress in enacting change. The second initiative, the PULSE Recognition Program, involves completion of the aforementioned Rubrics and a site-visit by two Recognition Team members to provide external insights and suggestions to foster a department’s change process. Eight departments participated in the Recognition Program in 2014. An evaluation of the documents yielded from the Recognition Program review of seven of the eight departments and a comparison of Rubric scores from before and three years following the site-visits uncovered several common elements required for successful department level change. These elements include an institutional culture that values and supports excellence in teaching and learning with resources and infrastructure, a departmental emphasis on program and course level assessment, and, most importantly, a departmental champion who actively supports endeavors that enhance teaching excellence.

## Introduction

Multiple change efforts are underway to improve the success of students in science, technology, engineering and mathematics (STEM). In addition, the body of knowledge regarding how people learn is expanding and high impact practices to support enhanced learning have been identified [1–3]. These practices are often incorporated into individual courses and learning is measured at the course level. However, to promote a unified and sustained impact on student learning and STEM student success, a more systematic and departmental level approach may lead to a greater impact over a shorter period of time.

In 2009, a series of conversations began about the future of biology education encompassing the changes in the field of biology and the research on cognition, teaching and learning. These conversations involved faculty, students, and professional society representatives, and culminated in a meeting that same year hosted by American Association for the Advancement of Science (AAAS). The meeting generated the 2011 report, *Vision and Change in Undergraduate Biology Education*: *A Call to Action (Vision and Change)* [4]. The report summarized meeting discussions and included a number of recommendations [5] such as 1) undergraduate biology curricula should foster scientific literacy in the following core concepts: evolution; pathways and transformation of energy and matter; information flow, exchange and storage; structure and function; and systems; 2) core competencies should be addressed in areas including the process of science; the interdisciplinary nature of science and the integration of science into society; communication and collaboration; quantitative reasoning; modeling, simulation, computational and systems level approaches; as well as the ability to use large databases to analyze data. 3) Finally, the *Vision and Change* [4] document recommended that faculty adopt evidence-based practices in education to introduce their students to the core concepts and competencies and to assess their learning in these areas.

Many individual life sciences faculty have looked at ways to improve their pedagogy, knowledge of course design, as well as assessment of student learning. They have participated in initiatives, workshops and conferences to improve their teaching. The work of individual faculty is important for building knowledge about effective teaching practices and course-level assessment. However, this work may be insular and not fully represent a unified department agenda to affect change. The literature suggests that departments should be the unit of change [6–7]. Baldwin [8] confirms this by recognizing that true STEM education reform can only occur when initiated at the department level. Departmental level initiatives require the development of a shared vision that is inclusive of all perspectives. Sustainable change begins with institutional support for the cultivation of faculty-student interactions in the department. A combination of bottom up (work in the department) and top down (institutional support) interests better facilitates change because the change initiatives are supported by the institution’s resources and infrastructure [9].

Recognizing the importance of fostering change at the department level, the Partnership for Undergraduate Life Sciences Education (PULSE) was formed in 2012. Forty PULSE Vision and Change (V&C) Leadership Fellows, each of whom served or were serving as life sciences departmental chairs/deans at that time, were selected to effect change. The mission of PULSE focuses on a vision of enacting change at the departmental level, rather than working with individual faculty.

To guide departments to critically think about their degree of implementation of the *Vision and Change* [4] recommendations, one of the PULSE working groups, the Recognition Team (RT), developed the PULSE Vision & Change Rubrics (V&C Rubrics). The V&C Rubrics included a series of five rubrics consisting of a total of 66 criteria examining departmental progress in five different areas: 1) curriculum alignment with the recommendations of *Vision and Change* [4]; 2) faculty practice/faculty support; 3) assessment; 4) infrastructure; and 5) climate for change [10]. Since only the curriculum alignment rubric is specific for life sciences, the other four rubrics can by used by any STEM department for evaluation of their progress in implementing departmental change. By late 2015, approximately 50 departments/programs across the country had submitted either their partial or complete Rubric data to the PULSE Rubric Portal. Using this national data set, the Rubrics underwent a development process aimed at optimizing and supporting their validity [11]. The V&C Rubrics can be found on the PULSE community website (www.pulsecommunity.org).

The *PULSE Rubrics* have multiple potential uses. First, and most importantly, they are very useful for departmental self-assessment, to see where a department is on the path to implementing the principles outlined in *Vision and Change* [4]. They allow departments to clearly identify areas of strength and weakness—which is the first step in the development of strategic plans to address areas that need attention. Departments can also use the Rubrics to serve as a measuring stick for departmental progress over time. At a minimum, the Rubrics may stimulate important conversations among faculty in a department.

The PULSE V&C Rubrics are one of the few tools available to assess departmental change. However, the PULSE Leadership Fellows realized sustained change is fostered through a process of self-evaluation, reflection and ongoing conversations between departmental faculty and administrators. The PULSE Recognition Program was designed with multiple tiers to motivate additional change towards and provide extrinsic acknowledgement of achievement of the recommendations of *Vision and Change* [4]. This program was launched in 2014, with funding support from the National Science Foundation (award # 1350120). The PULSE community website was used as a venue for a call for applications. Over 70 departments applied and eight were selected. Departments were selected based on their self-reported progress towards implementation of the *Vision and Change* [4] recommendations. An equal number of departments from community colleges, liberal arts colleges, research intensive and regional/comprehensive institutions were included in the program. The departments were asked to complete the V&C Rubrics with justifications for their scores, and submit materials, such as course syllabi, curriculum maps, assessment tools, departmental vision statements, teaching philosophy statements, and CVs. Two members of the Recognition Team (RT) then conducted a two-day site visit for each department, with one RT member from the same institution type as the school receiving the visit. During the site visit, the two Recognition Team site visitors (RTSV) observed classes and met with students, department faculty, and deans. They also toured facilities, including classrooms, teaching and research laboratories and unique features of each program or campus such as field stations, dedicated student research space, and resource/study rooms for students. After the visits, each department was provided with a report summarizing the impressions of the RTSVs that highlighted the strengths and weaknesses of their program and provided them with recommendations for sustained change. After the eight visits were completed, the RT met to review all of the information gathered from the site visits and assigned each department one of five PULSE Progression Levels based upon their level of alignment with *Vision and Change* [12]. Of the eight departments, one achieved “PULSE Progression Level III: Accomplished”. Six departments achieved “PULSE Progression Level II: Developing” and one department achieved “PULSE Progression Level I: Beginning”. None of the departments in the pilot received the highest designation (Level IV: Exemplar) or lowest designation (Level 0: Baseline).

Since the completion of the Recognition Program, the RT has continued its work. Of particular interest is discovering the impact of the Recognition Program on the participating departments’ since the initial site visits. In this paper, we report our findings based on a qualitative review of documents generated as part of the Recognition Program process. These documents include the departments’ initial application to the Recognition Program, the report of findings from the Recognition site visit, and a follow-up survey administered two years after the visit. Also, PULSE V&C Rubric scores were compared before and after the Recognition Program as a way to measure change. Review of these data uncovered motivation for participating in the Recognition Program, the elements required for departmental change, and the barriers that impede change. This work aims to inform life sciences stakeholders at undergraduate institutions of the necessary ingredients required to foster changes that have been demonstrated to enhance undergraduate life sciences student learning, persistence, and retention at the department level. Although this study focuses on life sciences departments, the conclusions may also reflect other STEM fields due to similarities in the ways departments in higher education operate.

## Methods

### Sample

The eight departments that participated in the Recognition Program in 2014 completed an application composed of open ended questions and submitted V&C Rubric scores [11] as part of the Recognition process. Faculty consensus scores were sought for completion of the V&C Rubrics.

Seven of the eight departments submitted responses to a follow-up survey composed of open ended questions that was developed by the RT in Fall 2016. Three of the seven departments submitted V&C Rubric scores in Fall 2017.

The applications and follow-up surveys were completed by the department chairs or program coordinators from the different institutions upon consultation with department faculty. Of the seven departments that completed both the application and follow-up survey, three had changes in leadership since the initial Recognition process.

The seven departments featured in this article provided informed consent to be included in this study. The study was approved by the IRB of Pace University (18–101) which stipulated that institutions could not be named, identified by their institution type or be identifiable in any way through the data presented herein. This limitation guided the manner in which the data were presented in this work and the seven institutions approved the data presentation prior to the submission of this work.

### Qualitative data analysis

A qualitative data analysis of the Recognition Program applications, RT reports to the departments, and follow-up surveys for each of the seven institutions that participated in this study was conducted by two members of the RT that did not participate in the selection of the departments for the program nor, the site visits or creation of the reports. The reports were each written generically by the RTSV based on their observations at the time of the visits and the qualitative data analysis to identify emergent themes from the reports was completed three years after the reports were generated. Emergent themes were identified using a grounded theory approach [13]. For each set of documents, the two RT members responsible for data analysis independently read and reviewed the documents and recorded emerging themes. The RT reports to the departments were evaluated twice–once to determine themes related to department/program strengths and once to determine themes related to department/program weaknesses. Then, the themes were discussed and a codebook of common themes agreed upon by both reviewers was established (Tables 1–4). Each researcher used the codebooks to re- analyze the stated documents for evidence of each theme. After the re-coding, the researchers met again to resolve any coding differences. Each data set was discussed so that the results had high levels of agreement [14]. The inter-rater reliability, by percent agreement, between researchers after code resolution was 99.5%. The data reported in Tables 1–4 represent themes that were explicitly identified as strengths or weaknesses in the reports analyzed for this study. If a particular theme was not found in the documents analyzed, the values in the Table do not add up to a total of seven. For each theme reported in Tables 1–4, we report only examples that included evidence from four of the seven (57%) or more of the participating departments.

**Table 1:** Analysis of the Recognition Team Site Visit (RTSV) reports: Institutional support that fosters department level change. Seven RTSV reports were analyzed for emergent themes. Three major themes were identified (A,B,C). Under each theme, categories were delineated.

**A. RESOURCES**		
**Resource Type Categories**	**Described in the RTSV Report as Area of Strength**	**Described in the RTSV Report as Area of Weakness**
Travel funds for teaching and learning professional development	4/7 (57%)	3/7 (43%)
Funding support for undergraduate student research	4/7 (57%)	0/7
Well-equipped teaching and research labs	4/7 (57%)	0/7
Strong support staff	3/7 (43%)^1^	4/7 (57%)^1^
Support centers/offices present at institution	5/7 (71%)	0/7
**B. INFRASTRUCTURE**		
**Infrastructure Type Categories**	**Described in the RTSV Report as Area of Strength**	**Described in the RTSV Report Reported as Area of Weakness**
Innovative classroom spaces that support learning for the number of students enrolled	1/7 (15%)	4/7 (57%)
Up to date research facilities	5/7 (71%)	0/7
**C. CHAMPIONS FOR CHANGE**		
**Champion Type Categories**	**Described in the RTSV Report as Area of Strength**	**Described in the RTSV Report Reported as Area of Weakness**
Chair/Program Director	5/7 (71%)	0/7
Dean	5/7 (71%)	1/7 (15%)

^1^ These percentages include one of the seven departments that had strengths and weaknesses in this same category.

**Table 2:** Analysis of the Recognition Team Site Visit (RTSV) reports: Departmental practices that support teaching and learning and foster department level change. Seven RTSV reports were analyzed for emergent themes. Four major themes were identified (A,B,C, D). Under each theme, categories were delineated.

**A. FACULTY HIRING PRACTICES**		
**Hiring Practice Categories**	**Described in the RTSV Report as Area of Strength**	**Described in the RTSV Report Reported as Area of Weakness**
Low number of adjunct, part time (TA/GA) and non-tenure track faculty	1/7 (14%)	3/7 (43%)
Explicit commitment to faculty diversity	2/7 (29%)	2/7 (29%)
**B. TEACHING ASSIGNMENTS**		
**Teaching Assignment Category**	**Described in the RTSV Report as Area of Strength**	**Described in the RTSV Report Reported as Area of Weakness**
Full time faculty teaching loads are 3+3 or less (with loads being lower for new junior faculty)	4/7 (57%)	0/7
**C. TEACHING AND LEARNING PROFESSIONAL DEVELOPMENT OPPORTUNITIES ARE AVAILABLE AND SUPPORTED**		
**Professional Development Opportunities Categories**	**Described in the RTSV Report as Area of Strength**	**Described in the RTSV Report Reported as Area of Weakness**
Internal opportunities available	5/7 (71%)	0/7
Support for external professional development	4/7 (57%)	0/7
Professional development focusing on teaching and learning is valued for tenure and promotion	0/7	4/7 (57%)
**D. COMMUNICATION AND BUY-IN WITHIN THE DEPARTMENT**		
**Level of Communication and Engagement Categories**	**Described in the RTSV Report as Area of Strength**	**Described in the RTSV Report Reported as Area of Weakness**
There is a core group of faculty who are passionate about and engaged in best practices in undergraduate education and student learning	5/7 (71%)	1/7 (14%)
There are formal and/or informal mentoring relationships with new faculty in the department to support teaching and learning efforts	5/7 (71%)	1/7 (14%)

**Table 3:** Analysis of the Recognition Team Site Visit (RTSV) reports: Adoption of best practices that foster department level change. Seven RTSV reports were analyzed for emergent themes. Five major themes were identified (A,B, C, D, E). Under each theme, categories were delineated.

**A. ADOPTION OF ACTIVE LEARNING STRATEGIES IN THE CLASSROOM**		
**Active Learning Practice Categories**	**Described in the RTSV Report as Area of Strength**	**Described in the RTSV Report Reported as Area of Weakness**
General statement that active learning strategies are incorporated into courses	5/7 (71%)	0/7
Specific examples of active learning strategies observed	6/7 (86%)	0/7
Subset of faculty do not incorporate active learning into the lectures	No strengths reported	5/7 (71%)
**B. RESEARCH OPPORTUNITIES FOR UNDERGRADUATE STUDENTS**		
**Type of Research Opportunity Categories**	**Described in the RTSV Report as Area of Strength**	**Described in the RTSV Report Reported as Area of Weakness**
Undergraduate faculty mentored research experiences are available	4/7 (57%)	3/7 (43%)
CUREs are integrated into teaching laboratories	6/7 (86%)	1/7 (14%)
**C. ATTENTIVENESS TO DIVERSITY AND INCLUSIVENESS**		
**Diversity/Inclusiveness Initiatives Categories**	**Described in the RTSV Report as Area of Strength**	**Described in the RTSV Report Reported as Area of Weakness**
Course design and department-initiated programs consider diversity/inclusiveness	5/7 (71%)	2/7 (29%)
**D. UNIQUE CURRICULAR/EXTRA-CURRICULAR EXPERIENCES ARE AVAILABLE**		
**Type of Experience Categories**	**Described in the RTSV Report as Area of Strength**	**Described in the RTSV Report Reported as Area of Weakness**
High impact curricular experiences are offered in the department	4/7 (57%)	1/7 (14%)
Opportunities to engage in the department outside of the classroom (student clubs, undergraduate research journal, internal student poster sessions, co-op experiences)	3/7 (43%)	1/7 (14%)
**E. NATURE OF ASSESSMENT PRACTICES**		
**Types of Assessment Practices Categories**	**Described in the RTSV Report as Area of Strength**	**Described in the RTSV Report Reported as Area of Weakness**
There is an institutional culture of assessment (assessment office, institutional drive for assessment)	2/7 (29%)	2/7 (29%)
There is an ongoing, well defined program assessment in place in the department	0/7	7/7 (100%)
Student learning outcomes and aligned assessments have been developed for individual courses	4/7 (57%)	1/7 (14%)
Instructor-independent, validated assessments are used	4/7 (57%)	0/7

**Table 4:** Themes from qualitative analysis of follow-up surveys responses from the departments.

**Question 1: Please choose the statement that best characterizes your current efforts to implement change in your department:** a. We are in the planning phases of implementing our change efforts. b. We have encountered a roadblock which has stalled our initial change implementation. c. We are currently implementing plans for change within the department. d. We have implemented some changes and are working on revising these efforts or starting new ones.	
**THEME**	**PERCENT**
Answer c: We are currently implementing plans for change within the department.	1/7 (14%)
Answer d: We have implemented some changes and are working on revising these efforts or starting new ones.	6/7 (86%)
**Question 2: If change has occurred, please describe the changes that occurred as a result of engagement with the PULSE V&C Recognition Program and describe the key elements in making change happen.**	
**THEME**	**PERCENT**
Theme 1: Developed courses/programs more aligned with *Vision and Change*	4/7 (57%)
Theme 2: Developed/revised course assessments	3/7 (43%)
Theme 3: Updated courses/created new courses to include evidence-based teaching practices	5/7 (71%)
Theme 4: Enhanced communication between faculty	3/7 (43%)
Theme 5: Increased faculty development opportunities	3/7 (43%)
**Question 3: If desired changes have not yet occurred, what barriers have prevented or slowed the desired changes and what would be needed to overcome these barriers?**	
**THEME**	**PERCENT**
Theme 1: Time	5/7 (71%)
Theme 2: Incentives/budget	2/7 (29%)
Theme 3: Technology	1/7 (14%)
Theme 4: Need help with developing assessment and/or analyzing assessment data	2/7 (29%)
**Question 4: Are your department Chairs/Program Directors or upper administrators supportive and involved, and what roles have they played?**	
**THEME**	**PERCENT**
Theme 1: Chairs/Program Directors involved	7/7 (100%)
Theme 2: Upper administrators involved	6/7 (86%)
**What roles have they played**	
**THEME**	**PERCENT**
Theme 1: Attended workshops/retreats	2/7 (29%)
Theme 2: Directly involved in change process	3/7 (43%)
Theme 3: Financial or infrastructure support	4/7 (57%)
**Question 5: Since your engagement with PULSE have you or any of your colleagues been motivated to participate in any professional development activities to enact the changes you want to see in your transformed department.**	
**THEME**	**PERCENT**
Yes	7/7 (100%)
**Question 6: If YES, please list the activities (open ended question)**	
**THEME**	**PERCENT**
Participated in internal professional development activities	3/7 (43%)
Participated in external professional development activities	3/7 (43%)
Applied for grant funding to support teaching and learning	4/7 (57%)
Enhanced or stimulated networking with faculty from other institutions	3/7 (43%)
Developed discipline-based educational research projects to assess classroom changes	3/7 (43%)

### PULSE V&C rubric scores

All eight institutions submitted consensus scores for the PULSE V&C Rubrics via the Rubric Portal once they were selected for the Recognition Program in 2014. In 2017, the RT requested institutions submit scores for the PULSE V&C Rubrics a second time. For comparison, we describe percent reported scores for each of the five rubrics. Percent reported scores were calculated by dividing total possible score for each of the five rubrics by the raw, unweighted scores the institution reported for each of the rubrics x 100 [11].

## Results

### Analysis of recognition program applications

#### Motivation for participation in the PULSE recognition program

The departments were selected for the Recognition Program because their application indicated they had made some initial changes in aligning their programs with the recommendations in the *Vision and Change* report [11]. Five of the seven (71%) departments indicated that they had implemented changes at the course level to align with *Vision and Change* [4]. Four of the seven (57%) departments also revealed that they had begun to implement the recommendations in *Vision and Change* [4] at the program level, as well. This information can be corroborated by the departments’ scores on the V&C Curriculum Alignment rubric. Brancaccio-Taras et al. [11] demonstrated that, generally, departments scored the highest on the Curriculum Alignment rubric.

The Recognition Program application asked the departments to describe their motivation to participate in the Recognition Program. A thematic review of the applications revealed five themes. More than half of the departments requested advice and feedback in order to further align their program and courses with *Vision and Change* (4/7, 57%) [4]. Additionally, some departments acknowledged that their participation in the Recognition Program would enable them to become leaders in national reform efforts (4/7, 57%). One department had an upcoming accreditation review and wanted feedback prior to the review; one department wanted ideas to enhance student success and retention; and one department wanted to leverage the feedback from the RTSV for more resources from their institution. Overall, the responses suggested that the departments were interested in feedback and were willing to make additional changes to further align their programs with the recommendations of *Vision and Change* [4].

### Analysis of recognition program reports

Each department received a written report following the Recognition site visit. The report included an executive summary, program overview, and a section on strengths as well as a section on weaknesses. A thematic analysis of the reports received by the seven departments identified the following three broad characteristics influencing their ability for change: (1) strong institutional support, (2) departmental practices that support teaching and learning, and (3) widespread adoption of evidence-based teaching and learning practices.

#### Characteristic influencing change 1- institutional support

For change to occur within a department, the institution must support it. We identified three main areas of institutional support in our coding of the reports that appeared to be essential for departmental change (Table 1). The first two, resources (Table 1A) and infrastructure (Table 1B), influence the physical working space and environment of the department. The third component, an administrative “champion” (Table 1C), is required to inspire department personnel and students, keep lines of communication open between all stakeholders, and leverage departmental/faculty needs with the administration.

*Resources*: Resources can be supplied to a department by an institution in many ways. We have identified five types of resources (travel for teaching and learning professional development; funding for undergraduate research; well-equipped teaching and research laboratories; strong support staff; and presence of support centers) that can support a department in adopting the recommendations in *Vision and Change* (Table 1A) [4]. The *Vision and Change* [4] document highlights the need to provide faculty with professional development opportunities in scientific teaching, assessment and other areas important for the advancement of undergraduate biology education in order to effect change. It is therefore, not surprising that four of the seven (57%) departments provided faculty with travel funds to teaching and learning professional development workshops (Table 1A). In total, the departments reported that 44 faculty had attended professional development workshops prior to the site visits by the recognition team members. Of the three (43%) departments that the RTSV reported received no or little funding to support professional development, one department desired professional development funding to support adjunct faculty (full-time faculty did receive funds for professional development); one department had an external grant to support faculty attendance at professional development workshops; and one department did not financially support teaching and learning professional development workshops because teaching and learning was not valued as part of the tenure and promotion process (Table 1A).

Providing undergraduates with authentic research experiences is highly recommended in *Vision and Change* [4]. Four of the seven (57%) departments provided undergraduate students with supplemental resources to help them engage in research (Table 1A). These include stipends, travel funds for regional or national professional meetings, institutional events and documents that highlight undergraduate student research productivity and outputs, and student research clubs/organizations. Two of the three departments that did not provide supplemental resources for undergraduate research do offer undergraduate research experiences, either as a one on one mentoring or as a course-based (or course-embedded) undergraduate research experience (CURE; [15–19]) (Table 3B). The third department without support for undergraduate research required a high level of research productivity and, as such, prioritized graduate student and postdoctoral training.

The RTSV reported that four of the seven (57%) departments had well-equipped teaching and research laboratory spaces (Table 1A). Well-equipped laboratory spaces included up-to-date equipment, computers and software, physical models, and an ample stock of consumable supplies and reagents.

A strong support staff, which includes administrative assistants, budgetary control personnel, laboratory preparation technicians, safety coordinators, teaching assistants (TAs) and graduate assistants (GAs), is integral to supporting a department or program. The RTSV reported that three of the seven (43%) departments had strong support staff whereas four (57%) did not (Table 1A). However, the RTSV reported that one of the departments had strengths as well as weaknesses in this category (which is represented in the reported numbers in the Table 1A). This is because they had strong support staff in administrative areas but, were lacking laboratory preparation support staff altogether.

*Infrastructure*: The RTSV reported that most of the departments (5/7, 71%) had several support centers/offices to assist both student and faculty development (Table 1A). Offices to support faculty included centers for teaching and learning as well as assessment, inclusive education and institutional research offices. Student support offices include those for quantitative skills development, undergraduate research, as well as tutoring and advising centers. The RTSV reports did not indicate any departments with weaknesses in this area.

The make-up of the physical spaces at an institution can influence teaching and learning effectiveness. During the site visits, the RTSV were attentive to whether or not flexible teaching spaces, IT resources, active learning classrooms, open spaces for collaboration and facilities that blur the lines between teaching and research were available. They noted that only one of the institutions had facilities that met these criteria (Table 1B). The RTSV observed that four of the seven (57%) institutions did not have sufficient infrastructure to support teaching and learning excellence. Weaknesses were reported if an institution lacked flexible classroom spaces (meaning that they had traditional classrooms and/or tiered lecture halls), lacked enough space to support the number of students enrolled in their courses, and/or lacked IT resources in all classrooms. It is important to point out that two of the four departments were from institutions that had weaknesses with respect to their infrastructure were in the process of building new facilities that would address many of these deficiencies.

The RTSV also noted the state of the research facilities at each institution because of the importance of engaging undergraduate students in research [20–22]. They indicated that five of the seven (71%) departments had modern research facilities that supported undergraduate research (Table 1B). Although no weaknesses were reported in this area, one of the institutions that was not included in the previously mentioned 71% was in the middle of building a new facility.

*Champions for change*: The final component of institutional support that is important for change is the presence of an administrative “champion” who is able to move change forward by working with all stakeholders at the institution to motivate, support and inspire change. Five of the seven (71%) departments had a chair or program director who the RTSV considered champions (Table 1C). Even though the RTSV did not specifically mention in the reports that the other two chairs/program directors were champions, we feel it is appropriate to conclude that all seven chairs/program directors should be considered champions, because all seven applications were authored by the chairs/program directors. Although having the chair as a champion is important in driving change, most often it is not sufficient to ensure long-term and sustainable change. Support from the upper administration is also important to facilitate change. The RTSV reported that five of the seven (71%) deans at the institutions were champions for change and supported initiatives at the department level to enhance teaching and learning (Table 1C). The deans were able to provide the departments with the resources they needed to effect change (Table 1A) and advocated for acknowledging faculty efforts to enhance their teaching effectiveness during tenure, promotion and performance review processes. The RTSV indicated one of the institutions had a dean who was unable to provide the support required by the department to initiate major improvements in teaching and learning. This institution had a strong focus on research productivity and, as such, the dean was more inclined to provide resources and advocate for research needs.

#### Characteristic influencing change 2—Departmental practice that supports teaching and learning excellence

A departmental culture that values excellence in teaching and learning impacts the practices and activities engaged in by the department. Over time, these practices can influence change.

*Hiring practices*: The first area that the RTSV considered when looking at department culture was the hiring practices at the institutions (Table 2A). Full time faculty are often provided with more opportunities to develop their teaching skills to enhance the learning of their students at their institution than part time faculty [23]. However, the reality is that most institutions rely on adjunct, part-time, non-tenure track and teaching assistants/graduate assistants (TA/GAs) to teach many of the courses that are required for an undergraduate degree, such as introductory biology [24]. This was the case at the institutions that participated in the Recognition Program. The RTSV noted that only one of the seven (14%) departments had very few part time, non-tenure track, or TA/GAs teaching their courses. On the other hand, the RTSV observed that three (43%) of the departments had more part time, non-tenure track or TA/GAs teaching their courses than full time, tenure track faculty (Table 2A).

As the diversity of students entering college increases, the *Vision and Change* document [4] highlights the need to consider both the diversity of the faculty and how to be more inclusive in the undergraduate classroom to meet the needs of a more diverse student population. The RTSV noted that two of the seven (29%) schools indicated they are fully committed to ensuring that their faculty hiring practices encouraged diversity. Two other departments did not explicitly state that faculty diversity was an area of focus with respect to their hiring practices (Table 2A).

*Teaching assignments*: Faculty at most institutions have conversations about teaching assignments and balancing their teaching responsibilities with their other work-related obligations. Higher teaching loads make it difficult for faculty to engage in activities to support thoughtful course design and professional development. The RTSV reported that four of the seven (57%) institutions had teaching loads of three courses per semester or less during an academic year with lower loads for newer full-time, tenure-track faculty to allow them time to establish themselves at the institution.

*Teaching and learning professional development opportunities*: As previously mentioned, professional development in evidence-based, student-centered pedagogies is important for change [25]. With respect to the presence of a departmental culture that values excellence in teaching and learning, five of the seven (71%) institutions demonstrated support for this culture by providing faculty with internal opportunities to improve their teaching practices either through institution supported training or peer mentoring (Table 2C). Four of the seven departments (57%) encouraged faculty attendance at external teaching and learning professional development workshops (Table 2C); these same four provided funding for these activities (Table 1A). Conversely, four of the seven institutions (57%) did not place much value on teaching and learning professional development it is not considered essential for tenure and promotion at those institutions. However, one of these four institutions is an institution that funds and supports external teaching and learning professional development. The department at this particular institution was also credited by the RTSV with having a chair/program director who was a champion for teaching and learning excellence and encouraged teaching and learning professional development (Table 1C). Two of the other institutions that did not place much value on teaching and learning professional development for tenure and promotion belong to the group of institutions that offered internal opportunities for professional development but, no funds for external professional development (Tables 1A and 2C). Both had champions who support teaching and learning professional development (Table 1C). The final institution that did not place much value on professional development for teaching and learning in the context of tenure and promotion, did not have funding to support external professional development. However, they did have grants to support travel for professional development and did not offer internal professional development opportunities even though they had a champion in the department (Tables 1A, 1C and 2C).

*Communication and buy-in within the department*: Change cannot happen in departments unless faculty work together and discuss teaching, learning and student success. Additionally, to support change, a nucleus of faculty is needed who value teaching and learning excellence, even if other members of the department and/or institution are more cautious in embracing these values. The RTSV indicated that five of the seven (71%) departments had a core group of departmental faculty who worked to enhance their teaching effectiveness, were engaged in ongoing change in their classrooms, and who effectively inspired a few resistant faculty to attempt to make changes (Table 2D). The RTSV also noted that five of the seven (71%) departments provided formal or informal mentoring around teaching and learning to new faculty. One of the seven departments had a core group of faculty who valued teaching and learning excellence but they experienced resistance with respect to widespread adoption of best practices from other departmental faculty because of the pressures for research productivity. As a result, this department did not have a formal or informal teaching and learning mentoring program for new faculty in place (Table 2D).

#### Characteristic influencing change 3- Adoption of best practices

One of the highlights of the *Vision and Change* [4] document is a call to engage undergraduate biology students in the practice and process of science using student-centered methodologies. The document provides examples of how this can be accomplished. Generally, the departments had the most strengths with respect to adopting active learning strategies in their classrooms and, we postulate that this is because of the level of faculty buy-in in each department (Table 2D).

*Active learning*: The RTSV noted that five of the seven (71%) departments stated that a vast majority of their courses incorporated active learning strategies (Table 3A). In addition, six of the seven (86%) departments provided the RTSV with evidence of specific examples of techniques that had been adopted, such as use of clickers, flipped classroom experiences, case studies, multimedia resources, primary literature review, and team-based learning. In contrast, RTSV also observed that five of the seven (71%) departments did have a sub-set of faculty who do not incorporate active learning into their courses and relied strictly on lecture to transmit information (Table 3A).

*Research opportunities for undergraduate students*: As previously described, providing undergraduates with research opportunities is essential for their preparation as future life sciences professionals [4]. Four of the seven (57%) departments provided undergraduate students with resources (Table 1A) as well as opportunities to engage in faculty-mentored research (Table 3B). Undergraduate student-faculty mentored research experiences were not widely available in three (43%) departments (Table 3B). However, six of the seven (86%) departments did offer CUREs in their teaching laboratories (Table 3B). The one department that did not offer any faculty mentored research or CUREs for their undergraduates was an institution with a heavy focus on research productivity and working with graduate students as well as training post-doctoral students instead of undergraduates.

*Diversity and inclusiveness*: The institution’s faculty hiring practices with respect to diversity have been previously mentioned. Two of the seven (29%) departments indicated that they were committed to hiring a diverse faculty (Table 2A). However, according to the RTSV, five of the seven (71%) departments made strides in designing programs that considered diversity and inclusiveness (Table 3C). One of the two departments that had reported this as an area of weakness informed the RTSV that increasing diversity and inclusion was an institutional goal. The other department with this weakness did not have a diverse faculty or student body but made efforts to apply for grant funding to increase student diversity.

*Unique curricular and extracurricular experiences*: Providing undergraduate students with unique, high impact experiences [2], such as interdisciplinary introductory STEM courses, utilizing local field stations and external research facilities, as well as capstone, co-op, and service learning experiences, enhances the educational experiences afforded to them. The RTSV indicated that four of the seven (57%) departments provided such experiences to their students (Table 3D). Another subset used high impact experiences to engage undergraduate students outside of the classroom. These included student clubs, undergraduate research journals and student poster sessions. The RTSV observed that three of the seven (43%) departments provided their students with co-curricular options to increase their engagement with the department. One department offered very few high impact educational experiences to their students. Instead, they focused on creating engaging classroom experiences despite the requirement for high research productivity by the faculty. (Table 3D).

*Nature of assessment practices*: In order to determine the impact of departmental innovations on student success, departments need an assessment plan in place that aligns program outcomes to course outcomes and utilizes both instructor-dependent and independent assessment tools. A model assessment plan is iterative, evaluates students’ progress at various points during their academic career and uses data to inform curricular changes. Program and course assessment data should be used to justify additional changes at the course or program level [26]. Prior work [11] has demonstrated that V&C Rubrics Assessment rubric scores were the lowest of all of the rubric scores reported by a vast majority of the departments that completed them, including the departments that participated in the Recognition Program. Therefore, it is not surprising that the RTSV observed an overall weakness in assessment. The RTSV reported that only two (29%) of the departments had an institutional culture of assessment with coordinated efforts to develop program assessment plans that included the reporting of assessment data to faculty, other college constituents, and an institutional assessment office (Table 3E). Two institutions had no obvious culture of assessment. In addition, the RTSV noted that none of the departments had a fully articulated and ongoing program assessment plan in place. Thus, many of the RTSV recommendations to the departments focused around the development of program assessment plans (Table 3E).

The departments were more successful at adopting course level assessments and had aligned course outcomes with specific assessment instruments. The RTSV reported that four of the seven (57%) had aligned assessments with their course outcomes. One department had not yet made gains in this area but was sending faculty to professional development workshops to learn about assessment. Finally, four of the seven (57%) departments had adopted instructor-independent assessment tools for their courses. Instructor-independent assessment tools include published concept inventories and nationally administered exams. For those departments that had not adopted instructor independent assessment tools, the RTSV provided them with suggestions and ideas so they could do so (Table 3E).

### Analysis of recognition program follow-up surveys

Two years after the site visits (2016), the RT reached out to the departments and asked them to complete a follow-up survey to determine if they had made any additional changes based upon the feedback they received from the RTSV. The responses to each question on the follow-up survey were coded for themes and are found in Table 4. The departments learned from their experiences with the Recognition Program and all reported they had implemented changes (Table 4, Question 1) despite encountering some barriers. Changes included the development of courses/programs (4/7, 57%), teaching practices (3/7, 43%) and assessments (5/7, 71%) reflecting the recommendations in *Vision and Change* (Table 4, Question 2) [4]. As previously described, assessment was an area of weakness for the departments. It was encouraging to learn that they had made strides in this important area. Some departments indicated that communication between faculty was enhanced (3/7, 43%) and other departments indicated that more of their faculty had attended teaching and learning professional development workshops (3/7, 43%).

The departments reported that the most common barrier they experienced while attempting to incorporate the recommendations from the RTSV was time limitations (5/7, 71%; Table 4, Question 3). Two (29%) reported that there were few incentives or little budgetary support to implement change. One (14%) department reported that their lack of institutional technology hampered their change process; two (29%) admitted they needed more help with respect to developing assessments and analyzing assessment data.

The departments indicated that their chairs/program directors were all involved in facilitating change. This supports our claim of the importance of having administrative champions to enact change. Six of the seven (86%) departments reported that the deans and upper administration were also involved in the change process (Table 4, Question 4). The departments reported that the chairs/program directors and deans attended workshops and retreats with the department faculty (2/7, 29%), were directly involved in the change process (3/7, 43%), or provided resources or support to improve infrastructure (4/7, 57%).

Finally, all departments reported that there was an increase in the support for and faculty attendance at teaching and learning professional development activities (Table 4, Questions 5 and 6). Three of the seven (43%) departments reported that they had an increase in faculty participation in internal workshops and three (43%) departments reported that they had an increase in faculty participation in external workshops. Four of the seven (57%) indicated they had applied for grant funding to support teaching and learning endeavors within their departments. Three of the seven (43%) departments stated they had developed teaching and learning faculty networks with faculty from other institutions and three (43%) now have faculty who have engaged in discipline-based educational research to assess progress in their course.

### Case studies describing the impact of the PULSE recognition program on department level change

In addition to the follow-up survey, three of the seven departments completed the V&C Rubrics in Fall 2017 so that we were able to compare the reported scores before and after participation in the Recognition Program. Along with this comparison, three case studies highlighting departmental journeys in their pursuit to enact departmental change are described. These case studies provide context to the data described above.

#### Department one

In their application, the Biology Department at Department One indicated that they wished to participate in the Recognition Program in order to get feedback on their progress implementing the recommendations in *Vision and Change* [4]. They also indicated they wanted to use the feedback to help them design their new science building and to be recognized as leaders in national life sciences education reform efforts. In the follow-up survey, they restated that their initial reason for applying for the Recognition Program was to confirm that their curricular revisions in response to *Vision and Change* [4] were heading in a positive direction.

Department One also pointed out in their application that they had adopted many of the *Vision and Change* [4] recommendations and had a long tradition of incorporating inquiry based, student-centered learning into their courses. A considerable number of their faculty had participated in teaching and learning professional development workshops, institutes, and seminars. They indicated that they had course and program assessment plans in place and used several published, validated tools for their assessment.

The RTSV visited Department One in September 2014 and noted several strengths and opportunities for improvement. They confirmed that the Biology Department had indeed been successful in adopting many of the *Vision and Change* [4] recommendations articulated in their application. In their report, the RTSV acknowledged that, “*Overall*, *the Biology Department at Institution One offers an outstanding biology education*. *The Biology faculty are passionate about student learning*, *energetic in their offerings and approaches and generous with their time and wisdom*. *Faculty are committed to curricular innovation and student success*. *Impressively*, *we observed a consistently high level of student engagement in all of the classrooms we visited*. *Much of what is going on at Institution One can serve as a national model of Biology education based on the principles of* *Vision and Change*.”

The RTSV recommended that the Biology Department consider adding both a quantitative requirement, such as a statistics or computer science course, and a senior capstone seminar course. The RTSV noted the department offered many upper level courses that cater to faculty expertise but enrollment in these courses was low and there were few full-time faculty teaching the introductory courses. As such, RTSV suggested that faculty teaching assignments be re-evaluated to ensure full coverage of the introductory courses and develop strategies to increase enrollments in upper level courses. Finally, although the department had developed both course-level and program-level assessments, the RTSV suggested that the department adopt assessment tools that would allow them to evaluate gains in student learning at different time points during their tenure at the institution, using a pre-, mid-, post-test format. Since the department’s program assessment was on a ten-year cycle, it was recommended that the department consider shortening their evaluation period in order to capture and respond to assessment data within two or three year cycles.

In response to the feedback from RTSV, Department One indicated that they had instituted a standing Assessment and Goals Committee that used surveys and questionnaires to evaluate students as they progress through the curriculum. The institution hired a dedicated data analyst in the Center for Teaching and Learning and to assist with analysis of assessment data. The department has added more quantitative skills across their curriculum, but has not yet added a quantitative requirement to their program. The department appointed a temporary working group to discuss adding a capstone requirement to the curriculum. They drafted a proposal for the department but a departmental vote did not favor the addition of the capstone experience.

Since the Recognition Visit, several faculty in Department One presented and/or led workshops at several national teaching and learning professional development conferences. The Biology Chair was invited to meet with biology faculty members at a nearby institution to discuss experiences with education reform and the PULSE Recognition Program. Because of the endorsement from the Recognition Program, Department One has become a leader in undergraduate biology education reform, which was an initial goal stated for participation in the Recognition Program.

**Fig 1:**
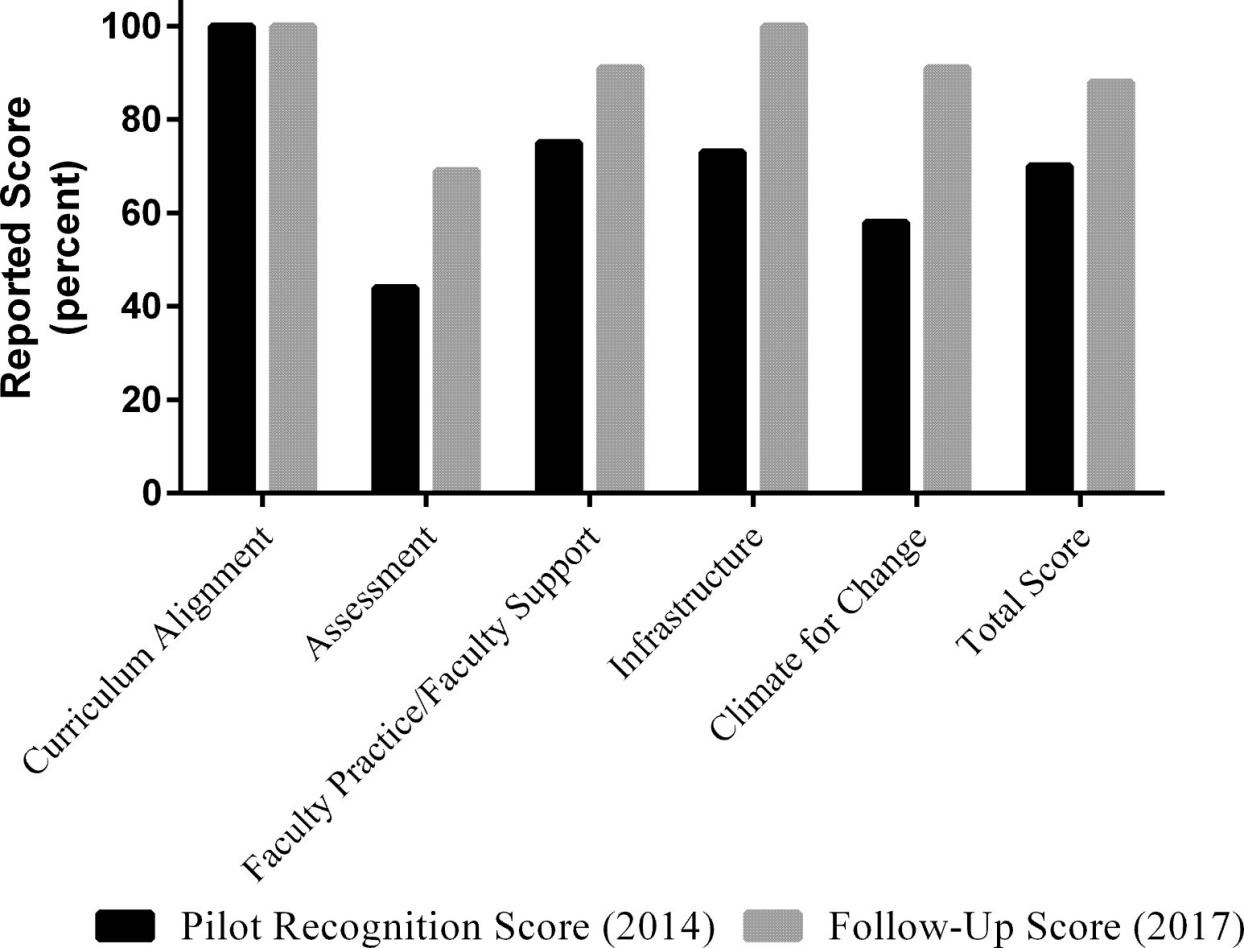
Comparison of pre- and post- V&C rubrics scores for department one. Department One completed the V&C Rubrics prior to the Fall 2014 Recognition Visit and then completed the V&C Rubrics in Fall 2017. Black bars represent the reported scores in percentage prior to the Recognition Visit and gray bars represent reported scores in percentage in the Fall 2018 follow-up.

As indicated earlier, departments participating in the Recognition Program were asked to complete the V&C Rubrics (Fig 1). The scores they reported on the rubrics were weighted as previously described [11]. Department One completed the V&C Rubrics again in Fall 2017. A comparison of the follow-up scores on the Rubric to the scores Department One reported prior to the recognition site visit appear in Fig 1. Department One was initially strong with respect to their adoption of the *Vision and Change* [4] core concepts and competencies (100%) and, as such, their scores did not change after the visit. However, scores on the other four rubrics (assessment, faculty practice/support, infrastructure and climate for change) did increase raising their mean total score from 70% to 88%. Of note, Department One was the weakest in areas of assessment (as assessed by both their answers on the Rubrics in 2014 and by the RTSV during the site visit) and, as described, they have made great gains in this area, with an average gain of 25%. Department One had a 16% gain on the Faculty Practice/Faculty Support rubric score, a 27% gain on the Infrastructure rubric score to 100%, and a 33% gain on the Climate for Change rubric.

#### Department two

In their recognition application, Department Two described an innovative program that they developed to enhance student retention and graduation rates. The program aims to provide students with opportunities for capstone undergraduate research experiences, mentoring, advising and tutoring support in a centralized center, and financial aid through scholarships. The department was motivated to participate in the Recognition Program in order to help improve their efforts to increase their retention and graduation rates. In the follow-up survey, they confirmed they were looking for an external evaluation from the RTSV to garner feedback on their program.

Department Two pointed out in their application that they partnered with an external, national organization to provide their students with undergraduate research opportunities and committed to developing a capstone Research and Measurements course, a more inquiry-based curriculum, and dedicated laboratory space with equipment for undergraduate research. The institution committed $80,000 to support faculty travel to professional development conferences to assist them in learning how to implement student-centered pedagogies for their courses. The institution also developed a centralized STEM student resource center that includes a director of undergraduate research, STEM student advisors, and tutoring services for STEM majors. Finally, NSF Scholarships in STEM (S-STEM) funding was received to provide scholarships to academically talented students with financial need.

The RTSV visited Department Two in September 2014 and noted several strengths and opportunities for improvement. In their report, RTSV acknowledged that, “*Overall*, *the Biology Program offers a strong education*, *especially considering the financial resources of the Institution*. *The Biology faculty are excited about teaching their students and invest a great deal of energy and enthusiasm as they implement best practices into their curriculum*. *Additionally*, *both the Chair and the Dean are fully committed to providing the students with an inquiry-based curriculum*, *containing authentic research experiences*, *which is an exceptional practice…* …*”*

The RTSV recommended that the department consider integrating systems biology as well as modeling and simulation competencies. The department offers numerous online laboratory experiences. The RTSV expressed concern about the quality of these experiences and suggested the department consider developing a self-study to compare student learning gains in the online versus students in the face-to-face laboratory courses. Although the RTSV complimented the assessment practices to evaluate student learning with respect to the general education outcomes in Department Two, they advised the department to consider adopting some validated, instructor-independent assessments at both the course and program levels. The department also teaches courses for pre-nursing students. The RTSV suggested that the faculty teaching the pre-nursing courses consider including more inquiry-based experiences as well as discussions about evolutionary biology in these courses.

In response to the feedback provided by the RTSV, the department claimed in the follow-up survey that it has been continuing to expand and revise their undergraduate research program. They reported that they are rewriting their student learning outcomes to align more closely with *Vision and Change* [4] and they held multiple meetings to develop and ensure modeling and simulation activities were included throughout their curriculum. In addition, the pre-nursing faculty added more active learning strategies into their courses. Finally, CUREs were added to the non-major’s biology courses and non-science faculty were included in the department’s CURE training.

At the time of the completion of the follow-up survey, several faculty were drafting an NSF IUSE grant to further support their retention initiatives. The retention program of Department Two also received a national award in 2016. Finally, faculty from Department Two have presented and/or led workshops at several national teaching and learning professional development workshops. According to Department Two, participation in the Recognition Program is one of the factors that has made them a leader in undergraduate biology education reform.

**Fig 2:**
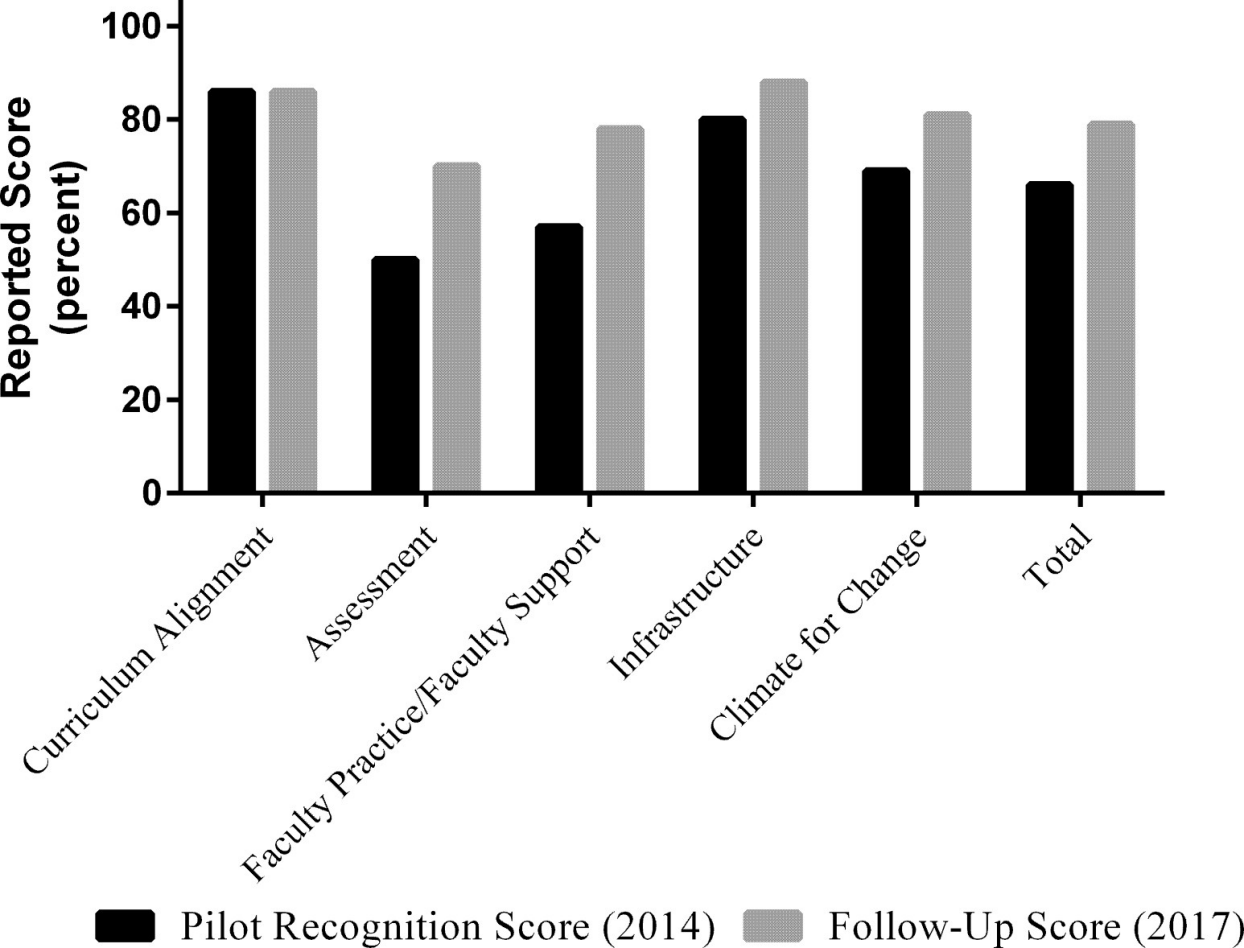
Comparison of pre- and post- V&C rubrics scores for department two. Department Two completed the V&C Rubrics in Fall 2014 and the V&C Rubrics in Fall 2017. Black bars represent the reported scores in percentage prior to the recognition visit and gray bars represent reported scores in percentage upon follow-up.

Follow-up scores for Department Two reported on the V&C Rubrics in 2017 were compared to the scores they reported prior to the Recognition Program visit (Fig 2). Department Two was initially strong with respect to their adoption of the *Vision and Change* [4] core concepts and competencies (86%) and, as such, their scores did not change over time. However, scores on the other four rubrics did increase raising their mean total score from 66% to 79%. Of note, Department Two was the weakest in areas of assessment, as reported by both their Rubrics scores in 2014 and the RTSV observations; however, they made great gains in this area. These gains were reflected in their follow-up Rubric scores with an average gain of 20%. Department Two had a 21% gain in reported score on the Faculty Practice/Faculty Support rubric, a gain of 8% on the Infrastructure rubric score, and a 12% gain on the Climate for Change rubric score.

#### Department three

In their Recognition Program application, Department Three indicated that the release of the V&C Rubrics and call for recognition participants were very timely as they had just completed major curricular changes to align their introductory courses to the recommendations in *Vision and Change* [4]. They also indicated that 18 of their faculty had attended the HHMI Summer Institutes (SI) and had incorporated the SI activities they developed into their individual courses. Finally, Department Three was undergoing reaccreditation the same year as the Recognition Program and believed the Recognition process would support their efforts in preparing for the reaccreditation. In the follow-up survey sent two years after the recognition visit, the Undergraduate Biology Program Director at Department Three confirmed that, “*The timing of the Certification process seemed ideal in terms of assessing whether the curricular changes and faculty development efforts were indeed leading to changes that align with Vision and Change*.”

The RTSV visited Department Three in May 2014. They noted several strengths and opportunities for improvement. In their report, RTSV acknowledged that, “*The Undergraduate Biology Program is a strong program with many faculty that value the success of undergraduate biology students…*.. *Many faculty view teaching as a rewarding and important part of their jobs and recognize the complementarity of teaching and research that is the fabric of an institution*.*”*

The Department Three Undergraduate Biology Program is comprised of faculty from three different departments who are coordinated through an executive committee of three senior faculty from each department and a program director. Students in the program enroll in three large enrollment lecture/laboratory introductory courses, each run by one of the three component departments. The courses are aligned to the recommendations in *Vision and Change* [4] and can be taken in any sequence. After completing the introductory sequence, students can specialize their upper-level coursework to satisfy their own areas of interest and career aspirations. A vast majority of the program’s courses engage students in the primary literature and most laboratory experiences are inquiry-based. The Program Director is a champion for the program and, according to the RTSV, has done an excellent job in keeping lines of communication open among all faculty and departments involved in the program. The RTSV noted that there was a strong culture and commitment to support undergraduate research with many resources available to couple students with mentors and to help students develop their research capabilities as they progress through the major. In addition, the program is well supported by other units at the institution including a STEM-based teaching and learning center, professional biology advisors, an undergraduate research office, a center for science communication and a center for inclusive education.

The RTSV recommended that the department consider developing a training program for the TAs and adjuncts who teach the introductory courses. At the time of the review, there was no such training in place and no method to evaluate the effectiveness of the TAs and adjuncts. Additionally, there were serious issues of coordination and alignment between the lecture and laboratory components of the intro course sequence. Thus, the TAs and adjuncts indicated that they were not clear about the teaching and learning outcomes for the labs. The introductory lecture course environment was also not favorable for student learning; enrollment in these courses was high and the learning spaces were very large (more than 500 seats), traditional, non-flexible tiered lecture halls. Future hiring would not be sufficient to staff smaller sections of the courses. Therefore, to ensure students are learning, the RTSV recommended that the introductory lecture course instructors incorporate more engaging, student-centered activities into their classes that were primarily lecture-based. Finally, the RTSV observed that, although the individual courses each had outcomes and aligned assessments, the program outcomes were less clearly defined. They suggested that the faculty invest time in aligning program outcomes to their course outcomes to ensure student growth as they progress through the curriculum.

In response to the feedback from the RTSV, the Program Director noted in the follow-up survey: “*One of the most major and immediate changes was a broader recognition of the value of a student centered view of what is happening in our classrooms by most instructional faculty*. *There was also a realization of a shared responsibility for the goals and outcomes of the Biology curriculum that extended beyond each faculty member’s commitment to their own course(s)*. *Although this understanding does not automatically result in tangible change*, *it lays an important foundation while also providing a compass to guide changes implemented at all levels*.”

A TA training program committee comprised of both faculty and graduate students was established in response to the RTSV’s feedback and they are working with the STEM teaching and learning center on campus to develop a mentoring program for the TAs and adjuncts. The process to develop a mentoring program has been slow and they noted that finding the time to commit to its development has been problematic. The introductory course faculty have worked to ensure that the course learning outcomes are fully aligned to *Vision and Change* [4], that there is more coordination between the introductory lectures and laboratories, and that more active learning experiences have been incorporated into the lectures. Of note, the Cell and Organ Physiology introductory course now includes a flipped component that was developed into a discipline-based education research (DBER) project. Several faculty have been invited to present their work at or have attended national teaching and learning professional development conferences. Department Three has reported that participation in the Recognition Program is one of the factors that has made them a leader in undergraduate biology education reform.

**Fig 3:**
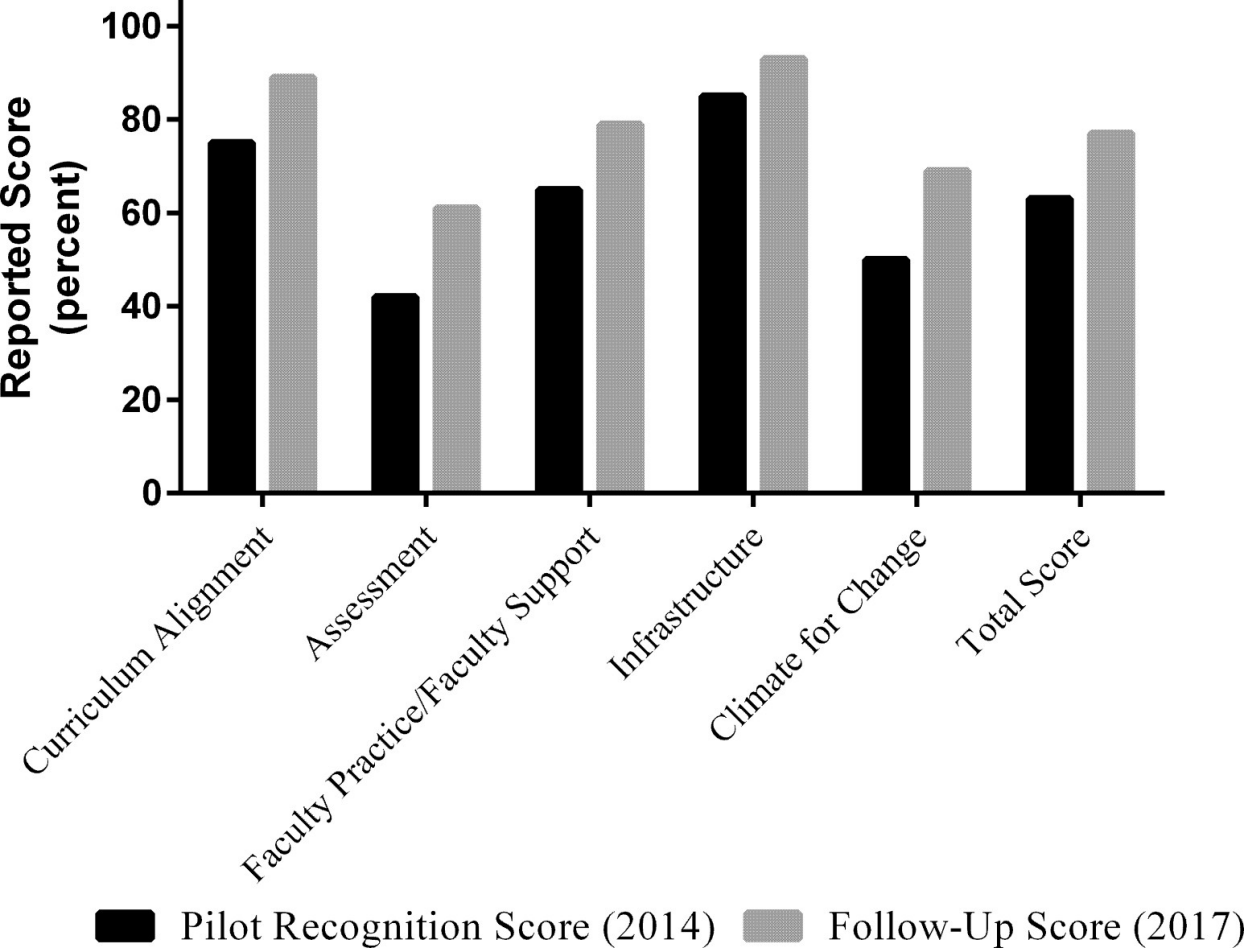
Comparison of pre- and post- V&C rubrics scores for department three. Department Three completed the V&C Rubrics in Fall 2014 and the V&C Rubrics in Fall 2017 as a follow-up. Black bars represent the reported scores in percentage prior to the recognition visit and gray bars represent reported scores in percentage upon follow-up.

A comparison of their reported follow-up scores on the V&C Rubrics in 2017 to the scores they reported prior to the recognition site visit appears in Fig 3. As a result of the recognition visit, the introductory faculty further aligned their courses with *Vision and Change* [4] and the scores on the Curriculum Alignment rubric increased from 75% to 89% (14% gain). The introductory course faculty also developed assessments that aligned with their course outcomes; this action explains the increase in the scores on the Assessment rubric from 42% to 61% (19% gain). As noted, the Recognition Program also resulted in a change in faculty attitudes about student centered learning and shared responsibility which may explain the increases on the Faculty Practice/Faculty Support rubric (14% gain) and Climate for Change rubric (19% gain) scores. There was an 8% increase on the Infrastructure rubric to a reported score of 93%, as well. The gains reported for the Infrastructure rubric are likely impacted by the new STEM facility that was constructed. Planning for that facility was underway while the RTSV were conducting the visit. Overall, the mean total rubric score increased from 63% to 77% with an average gain of 14%.

## Discussion

Based on our findings, a department positioned for sustained change would be strong in the three areas we have identified as broad characteristics that support change: support systems, departmental practices that support teaching and learning, and adoption of best practices in education (Tables 1–3). However, our data suggest that it is possible for a department to change even with weaknesses in some of these areas.

### Support systems, champions as driving agents for change

The RTSV defined a champion as an individual who serves as the driver for change in the department. The reports to the departments indicated that five of seven (71%) had a department chair or program director who acted as a champion. The reports also indicated that five of the seven departments (71%) had a champion in the dean’s office (Table 1). The significance of a champion to initiate and continue to support change cannot be understated. Initially, change agents encourage individuals or groups to reflect on their practices, particularly with regard to instruction, and make changes, leading to the identification of common experiences and interests as well as the development of new knowledge [27]. As these common experiences and interests emerge, a shared departmental vision can be established. The departmental vision has as its underpinning improving the student experience, which will lead to greater student success. The vision needs to be flexible, reflect the institution’s culture, include resources for program development, and once implemented, include the dissemination of measured results and next steps. [28].

Commonly, department chairpersons, deans or other college administrators are believed to be responsible for moving institutions towards new educational trajectories. However, the RTSV observed multiple leaders on campuses that enacted change. Departments often have a sub-set of faculty interested in improving the student experience and changing the way life sciences education is taught with the hope of improving student outcomes. Kezar, Gallant and Lester [29] describe grassroots leadership by faculty, fostering “bottom up” change initiatives as “tempered radicals” [29], faculty working outside the status quo to encourage change from the bottom up, since they truly have little to no formal authority. Usually, these change agents are able to work with individuals to increase awareness about new teaching practices. However, research has shown these efforts by change agents have led to new faculty learning about classroom innovations but has not led to faculty being supported to actually change their teaching. For implementation, a champion, careful monitoring, and self-reflection are also required [30].

### Departmental practices that support teaching and learning, faculty hiring practices effect change

Hiring practices and an institution’s reward system affect the degree to which faculty are willing to change their teaching methods. Many institutions rely heavily on the work of contingent faculty. Our analysis of the reports to the departments confirmed this. Only one department did not rely heavily on part-time/adjunct faculty, GAs/TAs, or non-tenure track faculty (Table 2).

Non-tenure track faculty account for 70% of all faculty members [31] Many of these instructors teach gateway STEM courses. Excellent introductory courses are critical for students so that they remain interested and master the basic core concepts and competencies required to be successful in advanced courses. It has been suggested that full-time tenured/tenure track faculty be assigned to teach introductory courses [32] as these faculty have more teaching experiences and may be more committed to the institution and its students. If it is not possible for full-time faculty to teach these important introductory courses, then contingent faculty need to be mentored by full-time faculty, included in departmental curriculum conversations, and provided with faculty development opportunities that focus on effective teaching strategies and their implementation.

### The adoption of evidence-based teaching practices in a department is not always enacted by all department faculty

The reports indicated that five of seven (71%) had a core group of faculty that support adoption of evidence-based teaching methodologies and five of seven (71%) had formal and informal teaching and learning mentoring programs for new faculty (Table 2), however, the reports also indicated that five of seven (71%) had a sub-set of faculty who did not incorporate active learning into their courses and strictly lectured to their students (Table 3). This is not surprising as not all faculty are knowledgeable regarding teaching techniques known to engage students’ interests and promote their learning. Due to this lack of knowledge, many instructors are unwilling to change their classroom practice. To overcome this barrier, sharing of resources among faculty has been shown to increase the use of these pedagogies of engagement [33]. However, unfortunately, some faculty know about these techniques, but simply do not employ them.

Throughout higher education, multiple instructional strategies implemented in classrooms across the country are categorized as active learning, student centered learning, collaborative learning, experiential learning and problem based learning; collectively referred to as transformational teaching [34]. It has been previously reported that multiple high impact practices need to be implemented to support student success, particularly with underserved students [35]. While multiple studies have examined the benefits of active learning, it is difficult to discern the actual amount of active learning being implemented in classrooms. Faculty have been known to either overestimate the amount of active learning versus lecture in their classrooms or implement these student-centered approaches in a way that makes them ineffective [36]. The use of institutional resources for faculty development is one way to address this problem. One successful faculty development model that relies on ongoing dialogue among faculty is faculty learning communities, [37]. It has been documented that biology faculty participating in learning communities have continued conversations about teaching and learning due to the collegial bond formed between group members, the low time commitment, and the positive interaction with a group facilitator [38].

For STEM disciplines, a strategy proven to positively impact students includes faculty mentored undergraduate research experiences [39] and course based undergraduate research experiences [15–19]. According to the reports, six of the seven (86%) departments incorporated course based undergraduate research experiences into their curricula (Table 3).

### Adoption of best practices, a culture of assessment

Institutions and departments with more full-time faculty are more likely to have the time for conversations about teaching, learning and student success. In addition, departmental plans resulting from ongoing discussions are more likely to be developed and accomplished. One important planning area is assessment of student learning as well as program assessment. Most of the departments in this study had assessment identified as an area for growth and improvement. Supporting this, the RTSV indicated that none of the departments had an ongoing, well defined program assessment plan in place in their departments (Table 3). Scott and Hawke [40] argue that external audits of higher education institutions serve as motivation for departments to document, review, and improve their actions. However, the prescriptive nature of program accreditation may be a disincentive to teaching innovations [41]. Therefore, it is important for departments to develop assessment plans and use the results to make informed decisions about course and program modifications to improve student learning. Departmental assessment plans that intentionally align stated outcomes with measures are more apt to generate data that promotes departmental dialogue and evidence for necessary changes. The implementation of an iterative assessment process creates a departmental culture of assessment within a department. In addition, linking a department’s assessment and evaluation processes to that of the institutions can generate transformation of the institution as a whole–along with its departments. [42].

Generally, the RTSV observed faculty buy-in for life sciences education reform as an essential element for departmental change. Change requires an investment of institutional resources. For example, research facilities as well as classroom spaces need to be kept up to date and faculty professional development needs to be supported so that high impact practices can be effectively implemented into life sciences classrooms. In the follow-up surveys, many of the departments reported that they had received resources because of the feedback from the RTSV (Table 4, Figs 1–3, and case study narratives). In addition, a department may have faculty who are truly interested in changing their teaching practices, and have committed time and energy to create interesting and engaging classroom activities for students. Their efforts alone will not generate departmental change. Anderson and colleagues [43] recommend engaging chairs, deans, and presidents so that the institutional culture and values, particularly of research universities, supports and rewards teaching excellence and the use of effective teaching practices. In addition, new teaching practices at different institution types need to be assessed and results reported to the scientific community as education research studies. Finally, hiring practices need to reflect a commitment to recruiting diverse faculty who also demonstrate excellence in teaching as well as research.

There were two limitations to this study. One limitation is the small sample size of seven departments. The departments that were selected to be part of the Recognition Program were selected, primarily, based upon their institution type to ensure that each institution type was represented in the Program. However, they were also selected because each department had provided the Recognition Team with some evidence that they had begun to embrace change–a second limitation to this study [11]. Despite these limitations, the literature on departmental transformation in the undergraduate STEM disciplines supports the conclusions drawn from this work. A hallmark of this study is the participation of variety of institution types including community colleges, liberal arts colleges, regional comprehensive universities and research universities. Our study found regardless of institution type, all departments in this study experienced challenges and difficulties in their efforts to foster change. In addition institutional culture rather than institution type is a major driver of change. The literature on departmental change in undergraduate STEM discipline supports our work. Although our findings result from the study of life sciences departments, the findings can be more broadly applied to other STEM disciplines.

Based on our analysis of the PULSE Recognition Program applications, post site-visit reports and follow-up surveys, for transformation of the educational practices life sciences departments to occur, an institutional culture that values excellence in teaching and learning and that seeks continuous development in these areas is essential. This institutional culture must support activities that promote teaching excellence for all faculty, tenured, tenure-track and adjuncts, so that student learning can be enhanced. In order to make conclusions about improvements in student learning, there must be an institutional emphasis on program and course level assessment, and assessment data must be used to inform decision making on both the departmental and institutional levels. Finally, a departmental champion who actively supports endeavors that enhance teaching excellence, and encourages communication between faculty and with administrators is an essential ingredient for change. A strong champion can foster the majority of faculty to actively buy-in to departmental change leading to improved learning outcomes for life sciences students. This will ultimately lead to enhanced life sciences student retention and persistence to graduation.

## References

[1] Ambrose S, Bridges M, DiPietro M, Lovett M, Norman M. How learning works: seven research-based principles for smart teaching. San Francisco: Wiley; 2010.

[2] Kuh GD. Excerpt from High-impact educational practices: What they are, who has access to them, and why they matter Washington, DC: Association of American Colleges and Universities 2008 Available from: www.aacu.org/leap/hips.

[3] National Research Council. How people learn: Brain, mind, experience, and school. Expanded edition Washington, DC: The National Academies Press 2000 Available from: 10.17226/9853.

[4] American Association for the Advancement of Science. Vision and change in undergraduate biology education: A call to action. 2011. Available from: http://visionandchange.org/files/2011/03/Revised-Vision-and-Change-Final-Report.pdf.

[5] Woodin T, Carter VC, Fletcher L. Vision and change in biology undergraduate education, a call for action–initial responses. CBE Life Sci. Educ. 2010; 9(2): 71–73. 10.1187/cbe.10-03-0044 20516350 PMC2879380

[6] Wieman C, Perkins K, Gilbert S. Transforming science education at large research universities: a case study in progress. Change. 2010;March/April: 7–14.

[7] Beach AL, Henderson C, Finkelstein N. Facilitating change in undergraduate STEM education. Change. 2012 Nov-Dec: 52–59.

[8] Baldwin R. The climate of undergraduate teaching and learning in STEM fields. New Direct Teach Learn. 2009;117: 9–17.

[9] Narum JL. The theory and practice of transforming STEM undergraduate education: reflections from the PKAL experience. Lib Educ. 2013;Winter: 26–31.

[10] Aguirre KM, Balser TC, Jack T, Marley KE, Miller KG, Osgood MP, et al The PULSE Vision & Change Rubrics. CBE Life Sci. Educ. 2013;12(4): 579–581. 10.1187/cbe.13-09-0183 24297283 PMC3846506

[11] Brancaccio-Taras L, Peteroy-Kelly M, Pape-Lindstrom P, Aguirre KM, Awong-Taylor J, Balser T, et al The PULSE Vision & Change Rubrics: A valid and equitable tool to measure transformation of life sciences departments at all institution types. CBE Life Sci. Educ. 2016;15(4)ar60. 10.1187/cbe.15-12-0260 PMC5132357 27856548

[12] Pape-Lindstrom P, Jack T, Miller K, Aguirre K, Awong-Taylor J, Balser T, et al PULSE Certification results. J. Microbiol. Biol. Educ. 2015;16(2): 127–129. 10.1128/jmbe.v16i2.974 26753017 PMC4690550

[13] Bryant A, Charmaz K. The SAGE handbook of grounded theory. Los Angeles: Sage Publications; 2007.

[14] Saldaña J. The coding manual for qualitative researchers. Los Angeles: Sage Publications; 2013.

[15] Awong-Taylor J, D’Costa A, Giles G, Leader T, Pursell D, Runck C, et al Undergraduate research for all: addressing the elephant in the room. Council on Undergraduate Research Quarterly 2016; 37(1): 11–19. 10.18833/curq/37/1/4

[16] Bangera G, Brownell SE. Course-based undergraduate research experiences can make scientific research more inclusive. CBE Life Sci. Educ. 2014;13(4): 602–606. 10.1187/cbe.14-06-0099 25452483 PMC4255347

[17] Brownell SE, Hekmat-Scafe DS, Singla V, Chandler Seawell P, Conklin Imam JF, Eddy SL, et al A high-enrollment course-based undergraduate research experience improves student conceptions of scientific thinking and ability to interpret data. CBE Life Sci. Educ. 2015;14(2)ar21. 10.1187/cbe.14-05-0092 PMC4477737 26033869

[18] Elgin SC, Bangera G, Decatur SM, Dolan EL, Guertin L, Newstetter WC, et al Insights from a convocation: integrating discovery-based research into the undergraduate curriculum. CBE Life Sci. Educ. 2016;15(2):fe2 10.1187/cbe.16-03-0118 27146158 PMC4909350

[19] Lopatto D, Tobias S. Science in solution: the impact of undergraduate research on student learning. Washington DC: Council on Undergraduate Research; 2010.

[20] Russell SH, Hancock MP, McCullough J. Benefits of undergraduate research experiences. Science 2007;316: 548–549. 10.1126/science.1140384 17463273

[21] Nichols ML. Faculty-mentored undergraduate research: A qualitative examination of its influence on student engagement and academic achievement. Ph.D. Thesis, The University of Pennsylvania. 2016. Available from: https://repository.upenn.edu/dissertations/AAI10158531

[22] Osborne KW, Woods KW, Maxwell WD, McGee K, Bookstaver PB. Outcomes of student-driven, faculty-mentored research and impact on postgraduate training and career selection. Amer. J. Pharm. Educ. 2018;82(4): 313–320. 10.5688/ajpe6246 PMC5972845 29867236

[23] Jacoby D. Effects of part-time faculty employment on community college graduation rates. J. Higher Educ. 2006;77(6): 1081–1102.

[24] Benjamin E. How over-reliance on contingent appointments diminishes faculty involvement in student learning. Peer Review 2012; Fall: 4–10.

[25] Whittaker JA, Montgomery BL. Cultivating institutional transformation and sustainable STEM diversity in higher education through integrative faculty development. Innov. High. Educ. 2014;39: 263–275.

[26] Towns MH. Developing learning outcomes and assessment plans at a variety of institutions: examples and case studies. J of Chem Educ. 2010;87(1): 91–96.

[27] Borrego M, Henderson C. Theoretical perspectives on change in STEM higher education and their implications for engineering education research and practices. 2010. Available from: http://homepages.wmich.edu/~chenders/Publications/2014BorregoJEEPerspectivesonChange.pdf

[28] Elrod S, Kezar A. Increasing student success in STEM: summary of a guide to systemic institutional change. Change: Mag High Learn. 2017;Jul-Aug: 26–34.

[29] Kezar A, Gallant TB, Lester J. Everyday people making a difference on college campuses: the tempered grassroots leadership tactics of faculty and staff. Studies Higher Educ. 2011;36(2): 129–151.

[30] Borrego M, Henderson C. Increasing the use of evidence-based teaching in STEM higher educational a comparison of eight change strategies. J Engin Educ. 2014;103(2): 220–252.

[31] Kezar A, Gehrke S. Why are we hiring so many non-tenure-track faculty? Liberal Education. 2014. Available from: https://www.aacu.org/publications-research/periodicals/why-are-we-hiring-so-many-non-tenure-track-faculty.

[32] Suchman EL. Changing academic culture to improve undergraduate STEM education. Trends Microbiol. 2014;22(12): 657–659. 10.1016/j.tim.2014.09.006 25449051

[33] McLaren HJ, Kenny PL. Motivating change form lecture-tutorial modes to less traditional forms of teaching. Austral Univer. Rev. 2015;57(1): 26–33.

[34] Slavich GM, Zimbardo PG. Transformational teaching: theoretical underpinnings, basic principles, and core methods. Educ Psychol Rev. 2012;24: 569–608. 10.1007/s10648-012-9199-6 23162369 PMC3498956

[35] Albertine S. Systemic change for student success: Goals and lessons of the LEAP states initiative. AAC&U Peer Review. 2011 Spring;4–8.

[36] Ebert-May D, Derting TL, Hodder J, Momsen JL, Long TM, Jardeleza SE. What we say is not what we do: Effective evaluation of faculty professional development programs. BioScience. 2011;61: 550–558.

[37] Cox M. Faculty learning communities: change agents for transforming institutions into learning organizations. To Improve the Academy. 2001;19: 69–93.

[38] McCourt JS, Andrews TC, Knight JK, Merrill JE, Nehm RH, Pelletreau KN, et al What motivates biology instructors to engage and persist in teaching professional development? CBE Life Sci. Educ. 2017;16(3)ar54 10.1187/cbe.16-08-0241 28821539 PMC5589434

[39] Lopatto D. Survey of undergraduate research experiences. CBE Life Sci. Educ. 2004; 3: 270–277. 10.1187/cbe.07-06-0039 PMC2104507 18056301

[40] Scott G, Hawke I. Using external quality audit as a lever for institutional change. Assess Eval Higher Educ. 2003;28(3): 323–332.

[41] Harvey L. The power of accreditation: views of academics. J High Educ Policy Manage. 2004;26(2):207–223.

[42] Jovanovic J, Armstrong MA. Mission possible: empowering institutions with strategies for change. Peer Rev. 2014;Spring: 21–24.

[43] Anderson WA, Banerjee U, Drennan CL, Elgin SCR, Epstein IR, Handelsman J. Changing the culture of science education at research universities. Science. 2011;331: 152–153. 10.1126/science.1198280 21233371

